# Astragalus injection protects cerebral ischemic injury by inhibiting neuronal apoptosis and the expression of JNK3 after cerebral ischemia reperfusion in rats

**DOI:** 10.1186/1744-9081-9-36

**Published:** 2013-10-01

**Authors:** Guangyi Liu, Jinming Song, Yunliang Guo, Tingting Wang, Zhen Zhou

**Affiliations:** 1 Institute of Cerebrovascular Diseases, Affiliated Hospital of Qingdao University Medical College, Qingdao, Shandong 266003, China

**Keywords:** Astragalus injection, Cerebral ischemia, Reperfusion injury, Apoptosis, JNK3, Rats

## Abstract

**Background:**

Astragalus is a widely used traditional Chinese medicine and has been proven beneficial for many aspects of human health. It is important to explore the neuroprotective effect and mechanism of astragalus injection in cerebral ischemia reperfusion injury.

**Methods:**

The focal cerebral ischemic model with middle cerebral artery occlusion (MCAO) reperfusion was established by Longa’s method in healthy adult male *Wistar* rats, and treated by injecting intraperitoneally astragalus injection (3 ml/kg). The neurobehavioral function of rats was evaluated by Longa’s test. The cerebral blood flow (CBF) was measured by laser Doppler flowmetry and the cerebral infarct volume was calculated by tetrazolium chloride (TTC) stain. The shape and structure of neurons in parahippocampal area was observed by HE stain and the neuronal apoptosis was detected by terminal deoxynucleotidyl transferase mediated dUTP nick end labeling (TUNEL) and flow cytometry. The expressions of c-jun N-terminal kinase 3 (JNK3) mRNA and protein were determined by RT-PCR and immunohistochemical assay and Western blotting respectively.

**Results:**

After treatment with astragalus injection, the expressions of JNK3 mRNA and protein reduced significantly, the number of neuronal apoptosis minus, the cerebral infarct volume shrink, the neuronal shape-structure and animal neurobehavioral function improved significantly than those in model rats.

**Conclusions:**

It is suggested that astragalus injection could inhibit neuronal apoptosis, reduce infarct volume and improve neurobehavioral function by down-regulating the expression of JNK3 gene after cerebral ischemia reperfusion injury in rats.

## Background

Neuronal apoptosis in ischemic penumbra is one of the mechanisms of neuronal damage in ischemic cerebrovascular disease [1]. The c-jun amino terminal kinase (c-Jun N-terminal kinase, JNK) signaling is an important pathway of mitogen-activated protein kinase (MAPK) [2] and plays an important regulatory role in programmed cell death [3]. The encoding JNK genes found presently in mammalian cells included jnk1, jnk2 and jnk3, and their corresponding coding product JNK1 and JNK2 expressed widely, while JNK3 expressed only in brain, heart and testicular tissues [4]. JNK3 is the key signal in neuronal apoptosis during brain ischemia [5], and the JNK3 gene knockout not only reduce the c-jun phosphorylation but also protect the brain injury caused by cerebral ischemia/hypoxia [6], so that inhibiting JNK3 expression and neuronal apoptosis maybe one of important methods to prevent and treat cerebral ischemic injury [7]. Iijima et al. [8] reported that propofol (propofol was originally developed in the UK by Imperial Chemical Industries as ICI 35868. Wikipedia® is a registered trademark of the Wikimedia Foundation, Inc.) could get rid of free radicals in the body, improve cerebral blood microcirculation, and inhibit neuronal apoptosis in hippocampus caused by ischemia in rats [9]. Previous experiments shown some ingredients of traditional Chinese medicine have neuroprotctive role [10,11]. Qu et al. [12,13] reported that astragaloside, a main ingredient of astragalus injection, could decrease leukocyte infusion in cerebral ischemia reperfusion tissue, and reduce inflammatory reaction and brain edema. The research results of Ye et al. [14,15] indicated that astragalus injection could inhibit the apoptosis-associated gene JNK3 expression, but its neuroprotective mechanism is not very clear now [16,17]. This work aims further to study the influence of astragalus injection on the expression of JNK3 and neuronal apoptosis following cerebral ischemia reperfusion injury, as well as its neuroprotective mechanism.

## Methods

### Establishment of animal models

Total of 70 healthy adult male *Wistar* rats (SPF grade with 4 months age and 203-250 g body weight) were supplied by the Laboratory Animal Center of Qingdao Drug Inspection, the license number is SCXK (Lu) 20090007. The disposition on animals in the experiments are in accordance with the relevant provisions of the guidance to take care of the experimental animals issued by the Science and Technology Department of the People’s Republic of China (The Ministry of Science and Technology of the People’s Republic of China: *Guidance Suggestions for the Care and Use of Laboratory Animals.* ; 2006). Of which 20 rats (n = 20) were randomly selected as control group and the rest 50 rats were established focal cerebral ischemic models by inserting intraluminaly a monofilament suture into middle cerebral artery from external-internal carotid artery according to Longa’s methods [18]. After ischemia 2 h, the monofilament thread was withdrawn to re-perfuse blood flow, and the rats in control group was subjected the same surgical operation without inserting a monofilament suture. Core body temperature was maintained at 36–37°C using a electric homeothermic blanket during and after the operation, and the cerebral blood flow (CBF) in parietal cortex was continuously monitored with laser Doppler flowmetry (PeriFlux 5000, Sweden). The rat was considered as a successful model when its CBF reduced to 30% and Horner’s sign appeared with right forelimb flexing and circling rightward as running. Of which 10 rats died or unsuccessful models, and the rest 40 successful models were subdivided randomly into model group (n = 20) and treatment group (n = 20).

### Treatment

Astragalus injection (No. Z31020084) is provided by Chengdu Diao Jiuhong Pharmacy Co., Ltd. Each milliliter (ml) astragalus injection contained 1 mg astragaloside (molecular weight: 784.97, molecular formula: C_41_H_68_O_14_). According to the treatment dose reported by Liu et al. [19], the rats in treatment group were intraperitoneally injected astragalus injection (3 ml/kg or 3 mg/kg) at ischemia 2 h, while equal normal saline for rats in control and model groups.

### Evaluating indexes

#### Neurobehavioral function

After treatment 24 h with astragalus injection, the neurobehavioral function was evaluated according to Longa’s test [18]. Score 0: no behavioral deficiency; Score 1: the rat’s forelimb *flexioned* when its tail was lifted suspension; Score 2: lateral thrust resistance decreased (lateral thrust test positive) and forelimb flexioned when tail lifted; Score 3: the same as midrange behavior and circling spontaneously. The higher score, the severe neurological function deficiency.

#### Cerebral blood flow (CBF)

CBF of the lateral hemisphere (parietal cortex) was continuously monitored with laser Doppler flowmetry. The head of laser fiber was placed stereotactically (SN-3, Narishige, Tokyo, Japan) in the area known to be ischemia after MCAO by a stainless cannula (the right caudate anterior 18.0 mm, right 8.0 mm, and height 18.0 mm according to an atlas. The rat CBF was recorded by Perisoft ware and calculated by CBF index (CBFI%) = (CBF of ischemic side / CBF of contralateral side) × 100.

#### Cerebral infarct volume (CIV)

After treatment 24 h with astragalus injection, 5 rats in each group were randomly selected and anesthetized with 10% chloral hydrate 0.3 ml (300 mg/kg) to be sacrificed by cutting the neck. The brain was removed rapidly and take out completely to be cut into five coronal sections of 2 mm thickness backward from optic chiasma. Then the sections were immersed in 2% 2, 3, 5-triphenyltetrazolium chloride (TTC) at 37°C for 10 min. The normal brain tissue showed red and infarct tissue white. The CIV is determined by Adobe PhotoShop CS after taking a photograph and presented as the percentage (%) of (cerebral infarct area / contralateral hemisphere area) × 100.

*Histopathology*: After treatment 24 h with astragalus injection, 5 rats in each group were anesthetized with 10% chloral hydrate and perfused from heart with 200 ml of normal saline and 200 ml of 4% paraformaldehyde solution. The brain was removed and dehydrated by gradient ethanol, cleared by xylene, embedded in paraffin and cut coronally backward from optic chiasma into pieces of thickness 5 μm, then adhered to the glass slices, and finally stored at 4°C. Four paraffin sections from each rat were selected to stain with hematoxylin for 5 min, differentiated by 75% hydrochloric acid ethanol for 30s, and stained by acidification eosin for 1 min. The neuron morphology in parietal cortex was observed in four non-overlapping visual fields under light microscope and presented as degenerative cell index (DCI): the number of degenerative cells / total cells in the visual field (Mean ± SD).

#### Neuronal apoptosis

According to the instruction of TUNEL apoptosis detection kit (Wuhan Boster Biotech. Co. Ltd., China), four paraffin sections form each group were conventionally de-waxed and hydrated to dispose with 3% H_2_O_2_ for 10 min at room temperature, and then digested by proteinase K (1:200) for 10 min at 37°C. Mixing TdT (1 μl) and Dig-dUTP (1 μl) into labeling buffer (18 μl) and add on each paraffin slice to react for 2 h at 37°C. Add biotin anti-digoxin antibody (1:100) and react for 30 min at 37°C, then add SABC (1:100) to react for 30 min at 37°C and colored with HRP-DAB reagent kit (Beijing Tiangen Biotech. Co. Ltd.). The TUNEL positive cells showed orange-brown nuclei under light microscope (Olympus CK2, Japan). Under high magnification (400 times), four non-overlapping visual fields in hippocampal area were randomly selected from each section to calculate the neuronal apoptosis index (NAI). NAI = the number of TUNEL-positive cells / the number of total cells in the field. Mean ± SD.

#### Immunohistochemical assay

The rabbit anti-rat JNK3 antibody and immunohistochemical SP kit (Wuhan Boster Biotech. Co. Ltd.) were used to detect the expression JNK3 in parietal cortex. Referring to the instructions of kit, four sections form each group were de-waxed and hydrated to dispose 3% H_2_O_2_ for 10 min at room temperature. Added JNK3 antibody (1:100) to react for 2 h at 37°C and SP (1:100) to incubate at 37°C for 30 min, and colored with HRP-DAB. The JNK3 positive cells appeared brown cytoplasm and measured by UV spectrophotometer (Beckmann USA) in four non-overlapping visual fields in each section. The JNK3 expression intensity was presented by relative absorbance (*A*) index (RAI): the *A* value of JNK3 positive cells minus the background *A* value, Mean ± SD.

#### Flow cytometry

After treatment 24 h with astragalus injection, 5 rats in each group were anesthetized with 10% chloral hydrate and perfused from heart with 200 ml of normal saline. Then remmoved the whole brain and put in the distilled cool water, peeled off the vessel and membranous tissues, and removed left parahippocampal tissue weighting 100 mg. According to the instruction of Annexin V-FITC apoptosis detection kit (Nanjing Biotech. Co. Ltd.), flow cytometry analysis was operated by FACScan Calibur (BD Company, USA) within 1 h and the result of neuronal apoptotic ratio (NAR%) was calculated by CELL Quest program analysis.

### Western blotting

After treatment 24 h with astragalus injection, 5 rats from each group were deeply anesthetized and perfused with normal saline. The whole brain was frozen in liquid nitrogen at −80°C and separated left parahippocampal tissue weighting 100 mg to grind with RIPA lysis buffer 100 μl at 4°C. After ultrasonic slurry and centrifuged with 12000 r/min for 10 min at 4°C (Eppendorf 5801, Germany), the supernatant was collected to determine the total protein concentration by BCA-100 kit. Took protein sample 50 μg to subject to electrophoresis on 10% SDS-polyacrylamide gels (SDS-PAGE) and transferred onto PVDF membrane (Millipore, Bedford, MA, USA) using a semi-dry electrophoretic transfer system. The PVDF membrane were blocked for 1 h at room temperature and then incubated with the primary goat anti-rat JNK3 antibody (1:300) overnight at 4°C. Finally the membrane was exposed 2–4 min in X-ray box, developed an image for 40s, fixed 2 min, washed 5 min and dried at room temperature. The absorbance (*A*) value of each band was determined by Quantity One software. In the same specimen, the value of 3-glyceraldehyde-phosphate dehydrogenase (GAPDH) was also detected to calibrate the concentration of each target protein. The relative *A* value (RAV) was calculated as follows: the *A* value of JNK3 / the *A* value of GAPDH.

#### RT-PCR

The above frozen sample was homogenized in Trizol reagent (Life Tech, USA) using 1 ml of Trizol per 50 mg of tissue. Total RNA was extracted from the tissue according to the manufacturer’s protocol and calculated the purity by A260/280 nm spectrophotometer. Using RT-PCR kit (Invitrogen, USA, USA), cDNA was synthesized from total RNA (5 μg). JNK3 sense primer: 5’-CTG ATG CAG TGC ACG ATC TAC-3’, anti-sense primer: 5’-AGC GTC GTA CTA GAC GTT GCG AT-3’, the product: 197 bp. GAPDH sense primer: 5’-TAG TCT ACA TGC TGC AGT ACT ACT-3’, anti-sense primer: 5’-CGA CTT GAT GTT AGC GAG ATA TC -3’ the product: 225 bp. PCR cycling conditions with reference to the use of JNK3 mRNA kit (Stanta Cruz, USA), 95°C predegeneration for 3 min; at 94°C modified for 30s; 56°C for 30s. 72°C for 40s, for 30 cycle. Finally extension at 72°C for 3 min. The experiment is repeated 3 times with a 2% agarose gel electrophoresis of PCR products, and visualized under ultraviolet illumination by ethidium bromide (EB) staining. The *A* value of each mRNA band in the same gel was captured and determined through Scion analysis software. The relative *A* value (RAV) of JNK3 mRNA were presented as: the *A* value of JNK3 / the *A* value of GAPDH.

### Statistical analysis

The experimental results were adopted to the analysis of SPSS15.0 software (SPSS, Chicago, IL, USA). The experimental data was showed in (means ± SD), and we use single factor for analysis of variance between groups, LSD-*t* test for comparing in pairs.

## Results

### Neurobehavioral function

The neurobehavioral function of rats in control group was normal and Longa’s test was 0 scales. The Longa’s test scales of rats in model group were significantly higher than those in control group (*t* = 30.82, *P* < 0.01), while in treatment group decreased significantly than those in model group (*t* = 7.57, *P*<0.01) and also higher than those in control group (*t* = 27.30, *P* < 0.01) (Table 1).

**Table 1:** Longa’s test scales and CBFI of rats (Mean ± SD)

**Groups**	**n**	**Longa’s test scales**	**CBFI (%)**
Control group	20	0.00 ± 0.00	3.53 ± 1.13
Model group	20	2.25 ± 0.45^*^	73.25 ± 5.45^*^
Treatment group	20	1.553 ± 0.35^△#^	81.50 ± 6.30^△#^

Compared with control group, ^*^t = 30.26-30.82, *P* < 0.01; ^△^*t* = 18.10-27.30, *P* < 0.01; ^#^Compared with model group, *t* = 7.57-6.11, *P* < 0.01.

### Cerebral blood flow

The cerebral blood flow index (CBFI%) of rats in control group was 100, which in model group (73.25 ± 5.45) reduced significantly (*t* = 30.26, *P* < 0.01), while in treatment group (81.60 ± 6.30) was significantly higher than that in model group (*t* = 6.11, *P*<0.01) and also lower than that in control group (*t* = 18.10, *P* < 0.01) (Table 1).

### Cerebral infarct volume

**Figure 1:**
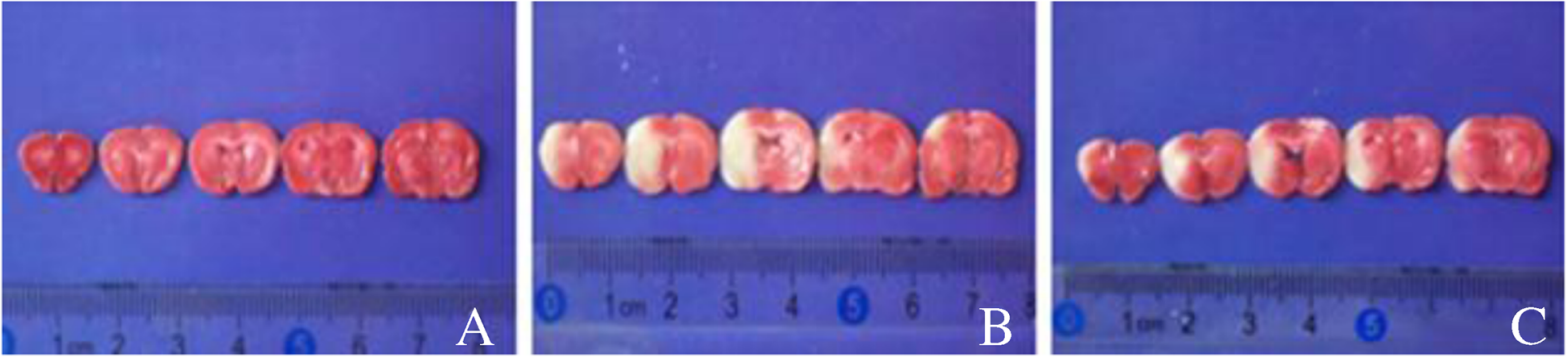
**The cerebral infarction volume of rats detected by TTC stain. A**. Control group, **B**. Model group, **C**. Treatment group

The normal brain tissues showed red and the infarct brain tissue showed white (Figure 1). No cerebral infarct area was found in the brain tissue of control group rats (CIV = 0), while a large infarct volume (CIV = 70.50 ± 6.75) existed in model group, and CIV reduced to 45.35 ± 5.62 in treatment group significantly (*t* = 8.11, *P*<0.01).

### The pathology in parahippocampal area

**Figure 2:**
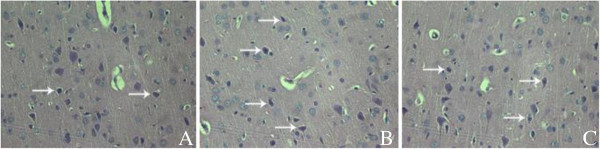
**The cellular shape and structure in parahippocampal area of rats, HE staining × 400. (A)** Control group: neurons arrange neatly, cell structure is completely. **(B)** Model group: neurons arranged disorderly, cytoplasm and nucleolus dyed deeply. **(C)** The number of degenerated cells (→) reduced than that of model group.

Hematoxylin-eosin (HE) staining (Figure 2) showed that the neurons in parahippocampal area arranged neatly, the cellular structure was completely, and the degenerative cell index (DCI) was 022 ± 0.07. In model group, the neurons degenerated seriously with triangular or irregular shape, the cytoplasm and nucleus concentrated, and the DCI (0.56 ± 0.20) was higher than that in control group (*t* = 4.54, *P*<0.01). In treatment group, the DCI (0.33 ± 0.14) was significantly lower than that in model group (*t* = 2.43, *P*<0.05) and also higher than that in control group (*t* = 2.35, *P* < 0.05).

### Neuronal apoptosis in parahippocampal area

**Figure 3:**
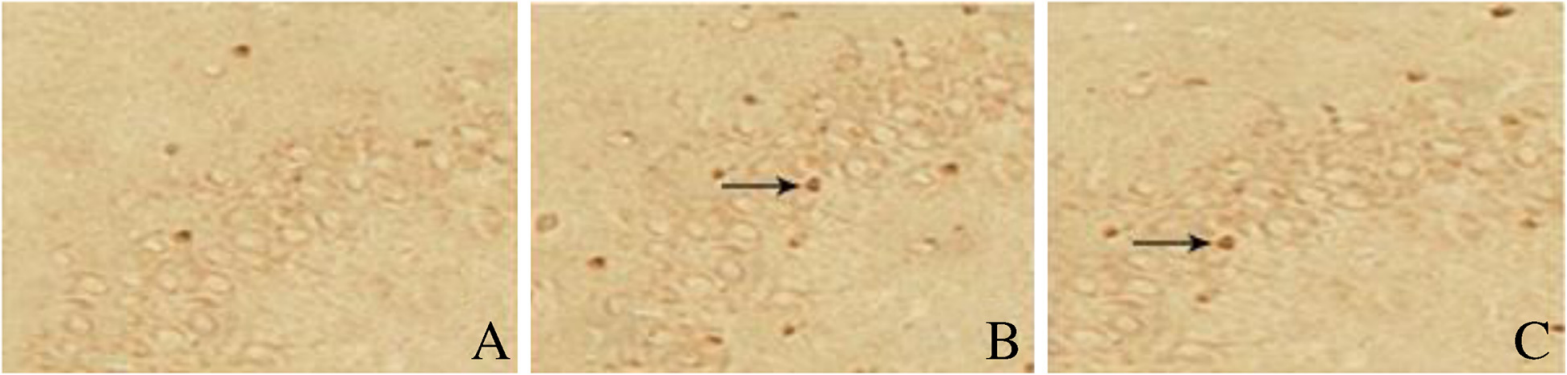
**Apoptotic neurons in hippocampal CA2 area detected by TUNEL × 400. A**. Control group, **B**. Model group, **C**. Treatment group. → apoptotic neurons

TUNEL staining (Figure 3) showed that a few apoptotic neurons existed in control group (NAI = 0.12 ± 0.02). The number of apoptotic neurons in model group (NAI = 0.33 ± 0.07) increased significantly than that in control group (*t* = 8.16, *P* < 0.01), while decreased significantly in treatment group (NAI = 0.26 ± 0.04) than that in model group (*t* = 2.46, *P* < 0.05), and also higher than that in control group (*t* = 8.86, *P* < 0.01).

**Figure 4:**
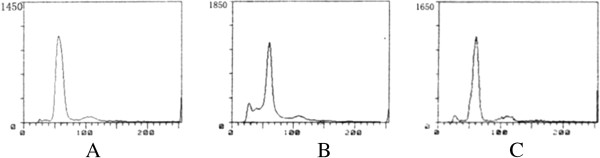
**Neuronal apoptotic ratio (NAR) in parahippocampal area detected by flow cytometry. A**: Control group, **B**. Model group, **C**. Treatment group

Flow cytometry (Figure 4) indicated that neuronal apoptotic ratio (NAR) in model group (6.56 ± 1.65) increased significantly than that in control group (1.67 ± 0.35) (*t* = 8.20, *P* < 0.01). In treatment group, the NAR (4.35 ± 1.12) decreased obviously than that in model group (*t* = 3.13, *P* < 0.01) and also higher than that in control group (*t* = 6.46, *P* < 0.01).

### The expression of JNK3

**Figure 5:**
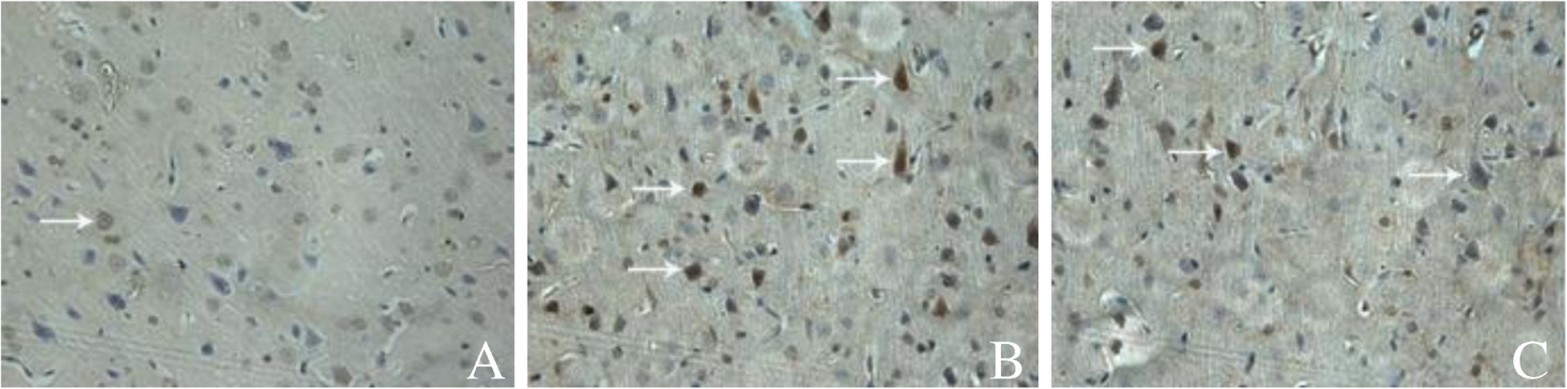
**The expression of JNK3 in parahipppocampal area, SP staining × 400. (A)** Control group: neurons arranged neatly, cell structure was completely. **(B)** Model group: neurons arranged disorderly, the number of JNK3-positive neurons increased significantly. **(C)** Treatment group: the number of JNK3-positive neurons (→) decreased significantly.

Immunohistochemical staining (Figure 5) showed that the expression of JNK3 in parahippocampal area of control group (RAI = 0.14±0.05) was slightly, which increased significantly in model group (RAI = 0.37± 0.12) than that of control group (*t* = 5.00, *P* < 0.01), while reduced obviously in treatment group (RAI = 0.26 ± 0.08) than that of model group (*t* = 2.15, *P* < 0.05) and also higher than that in control group (*t* = 3.59, *P* < 0.01).

**Figure 6:**
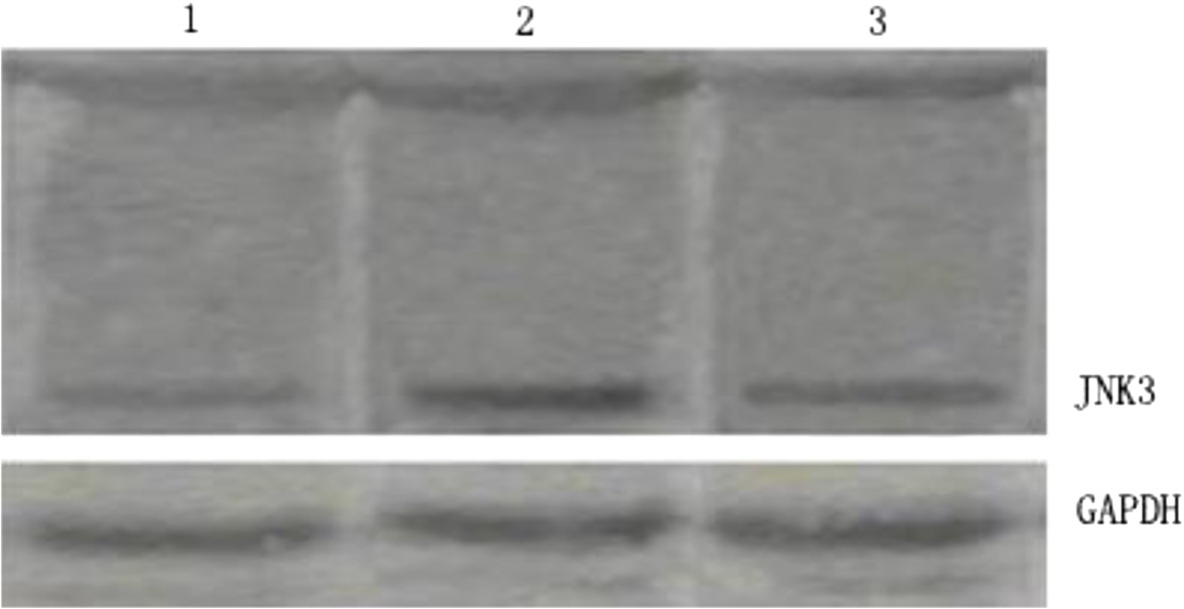
**The fragmentation formed by JNK3 protein detected by Western blotting.** Line 1: a weaker fragment was formed by JNK3 protein in control group. Line 2: a strong fragment was formed by JNK3 protein in model group. Line 3: a middle fragment was formed by JNK3 protein in treatment group.

Western bloting of quantitative analysis (Figure 6) indicated that JNK3 (11kD) expressed slightly in control group (RAV = 0.33 ± 0.08), and increased significantly in model group (RAV = 0.87 ± 0.21) than that in control group (*t* = 6.84, *P* < 0.01), while reduced obviously in treatment group (RAV = 0.56 ± 0.14) than that in model group (*t* = 3.54, *P* < 0.01) and also higher than that in control group (*t* = 4.04, *P* < 0.01).

### The expression of JNK3 mRNA

**Figure 7:**
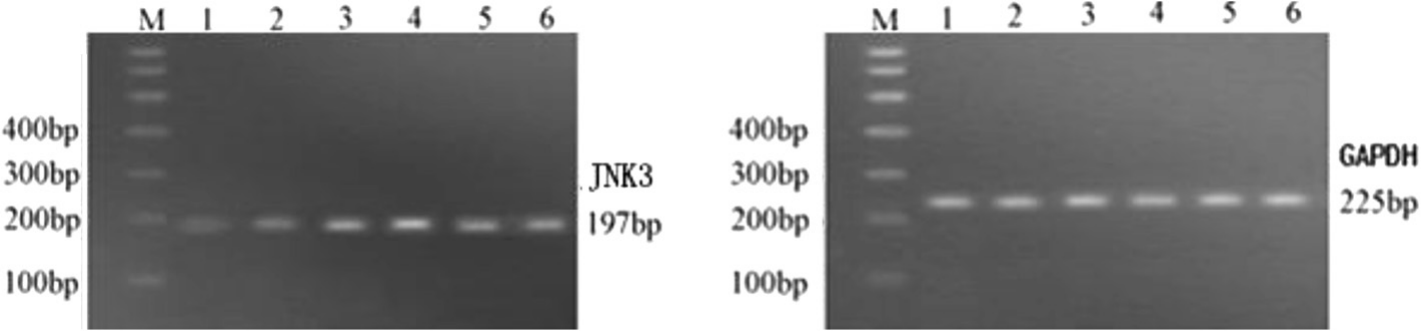
**Electrophoresis analysis of RT-PCR products of JNK3 mRNA (left) and GAPDH (right).** M: Maker; Line 1–2: weak light bands in control group; Line 3–4: strong light bands in model group; Line 5–6: middle light bands in treatment group

The relative *A* values of JNK3 and GAPDH presented the relative content of PCR products (Figure 7). The JNK3 mRNA in control group (RAV = 0.33 ± 0.07) expressed slightly and expressed significantly in model group (RAV = 0.93 ± 0.21) stronger than that in control group (*t* = 7.67, *P* < 0.01), while decreased obviously in treatment group (RAV = 0.67 ± 0.17) than that in model group (*t* = 2.72, *P* < 0.01) and also higher than that in control group (*t* = 6.26, *P* < 0.01).

## Discussion

Cerebral ischemia and reperfusion can induce the expression of apoptosis-related genes c-jun N-terminal kinase 3 (JNK3) to cause neuronal apoptosis [20]. It is the main purpose of treatment that re-canalizing the occluded artery early as soon as possible for the regional blood supply in ischemic penumbra to save neurons’ functions [21]. The main direction of the current treatment is how to prevent the delayed neuronal death to rescue ischemic penumbra [22]. JNK3 is located in the cytoplasm of neurons, which is activated by cerebral ischemia reperfusion injury and transferred into the nucleus, and then activates the other transcription factors c-Jun and Jun D. The activated transcription factors (e.g. c-Jun and Jun D) combined with *cis*-actingelement to cause a large number of gene expressions associated apoptosis, which related closely to delayed cell death [23-25]. Kuan et al. [5] reported that JNK3 is the key signal in neuronal apoptosis during brain ischemia, so inhibiting JNK3 expression conuld reduce neuronal apoptosis and treat cerebral ischemic injury [7]. Ren et al. [26] reported that astragalus could (1) eliminate free radicals and avoid lipid peroxidation to improve cerebral function, (2) reduce the content of excitatory amino acid and restrain the expression of heat shock protein 70 and alleviate the cerebral ischemic injury, (3) reduce the permeability of blood vessel and improve of hemorrheology after ischemia, (4) restrain the hypernormic expression of glial fibrillary acidic protein in early period of cerebral ischemia and inhibit neuronal apoptosis effectively. Ye et al. [14] reported that astragalus injection could inhibit neuronal apoptosis and expression of JNK3 after hypoxia/hypoglycemia and re-oxygenation in hippocampal neurons.

In this experiment, the results show that the expressions of JNK3 mRNA and protein increased significantly after cerebral ischemia reperfusion to induce neuronal apoptosis, neuronal degeneration and cerebral infarction, which caused disorder in neurobehavioral function disorderly. In model group, RT-PCR results indicated that JNK3 mRNA expressed significantly and immunohistochemical staining and quantitative Western blotting showed JNK3 protein was expressed more than in control group. At the same time, TUNEL assay and flow cytometry also showed neuronal apoptosis highly, and hematoxylin-eosin staining showed neuronal degeneration seriously with large ischemic infarction and neurobehavioral dysfunction of rats. These results further indicated that cerebral ischemia caused JNK3 expression to inducing neuronal apoptosis and nervous dysfunction. After treatment with astragalus injection, the expressions of JNK3 mRNA and JNK3 protein of parahippocampal area decreased significantly than those in model group, so reduced the JNK3 protein activity and inhibited neuronal apoptosis, and as a results, reduced the cerebral infarction volume and improved the neuronal structure and neurobehavioral function of rats. Our research results were coincident with the previous reported [27,28], which further suggested that astragalus injection could inhabit neuronal apoptosis in ischemic area, reduce the cerebral infarct volume and improve the neurobehavioral function of rats. It might be the molecular mechanism of astragalus reducing JNK3 expression and inhibiting neuronal apoptosis to recover neuronal structure and function of rats after cerebral ischemia reperfusion injury.

## Conclusions

Astragalus injection could reduce infarction volume and improve neurobehavioral function by reducing the expression of JNK3 gene to inhibit neuronal apoptosis following cerebral ischemia in rats.

## Abbreviations

MCAO: Middle cerebral artery occlusion; JNK3: c-jun N-terminal kinase 3; CBF: Cerebral blood flow; TTC: Triphenyltetrazolium chloride; NAI: Neuronal apoptosis index; TUNEL: Terminal deoxynucleotidyl transferase mediated dUTP nick end labeling.

## Competing interests

The authors declare that they have no competing interests.

## Authors’ contributions

LGY and GYL was responsible for design, experiment and manuscript writing. SJM, WTT and ZZ carried out the experiments, data acquisition and analysis. All authors read and approved the final manuscript.
